# Conviction under New Jersey’s New Veterinary Law

**Published:** 1903-05

**Authors:** 


### CONVICTION UNDER NEW JERSEY'S NEW VETERINARY LAW. WARNING TO OFFENDERS.

The first case tried under New Jersey's new law, Chapter 18, laws 1902, resulted in the conviction of the defendant.

R. R. Sample was arrested and held for the January Grand Jury of Monmouth County, which indicted him for practising veterinary medicine without a license in violation of the law. He was tried, convicted, and fined $100 and costs of court, bringing the amount up to $140. In default of payment he was sent to the Freehold jail, where he is still serving time.

Drs. Height, of Asbury Park, and Clark, of Long Branch, deserve special credit in this case. The Veterinary Medical Association of New Jersey is determined that the law shall be enforced, and offenders had better take warning.

The penalty that R. R. Sample is paying ought to be warning enough. We do not believe that it will be necessary to prosecute many cases in New Jersey.

